# Retinoblastoma binding protein-1 (RBP1) is a Runx2 coactivator and promotes osteoblastic differentiation

**DOI:** 10.1186/1471-2474-11-104

**Published:** 2010-05-28

**Authors:** David G Monroe, John R Hawse, Malayannan Subramaniam, Thomas C Spelsberg

**Affiliations:** 1 Department of Biochemistry and Molecular Biology, Mayo Clinic College of Medicine, Rochester, Minnesota 55905, USA

## Abstract

**Background:**

Numerous transcription factors are involved in the establishment and maintenance of the osteoblastic phenotype, such as Runx2, osterix and Dlx5. The transcription factor retinoblastoma binding protein-1 (RBP1) was recently identified as an estrogen regulated gene in an osteosarcoma cell model. Since the function of RBP1 in osteoblastic differentiation and mineralization is unknown, we investigated the role of RBP1 in these processes.

**Methods:**

To create a cell model with suppressed RBP1 expression, primary calvarial osteoblasts were infected with a shRNA lentiviral vector specific for mouse RBP1 (CalOB-ΔRBP1) or a scrambled control shRNA lentivirus (CalOB-Control). Stable cell lines were generated and their mineralization potential was determined using osteoblastic differentiation medium, Alizarin Red staining, and quantitative PCR (QPCR) analyses. Runx2 coactivation by RBP1 was determined through the use of transient transfection assays.

**Results:**

Stable expression of the RBP1 shRNA lentivirus in CalOB-ΔRBP1 cells resulted in a 65-70% suppression of RBP1 expression. Osteoblastic mineralization assays demonstrated that suppression of RBP1 results in a potent delay in osteoblastic nodule formation in the CalOB-ΔRBP1 cells with a concomitant decrease in the expression of the osteogenic transcription factors Runx2 and osterix, along with decreases in BMP2, alkaline phosphatase, osteocalcin and bone sialoprotein. Regulation of Runx2 expression by RBP1 was shown to be mediated through the proximal P2 Runx2 promoter. Furthermore, RBP1 was demonstrated to be a potent coactivator of Runx2 transcriptional activity on two known Runx2 reporter constructs. These data suggest that the expression and activity of Runx2 is critically dependent on the presence of RBP1.

**Conclusions:**

This study is the first to demonstrate that RBP1 is an important mediator of the osteoblastic phenotype and clearly defines RBP1 as a novel transcription factor involved in the well known Runx2-osterix transcriptional cascade. As such, the effects of RBP1 on these processes are mediated through both regulation of Runx2 expression and transcriptional activity.

## Background

Many transcription factors contribute to the establishment and maintenance of the osteoblastic phenotype, either directly or indirectly. Some well-known examples of such transcription factors are Runx2 and osterix. Deletion of either in mice results in a drastic phenotype where the cartilaginous skeleton fails to ossify, leading to a complete lack of bone [1-4]. Therefore, the identification and characterization of ancillary transcription factors, which regulate both the expression and transcriptional function of these master controller transcription factors (e.g. Runx2 and osterix), is essential for further understanding the process of osteoblastic differentiation and developing future therapeutic interventions to address disorders of bone metabolism.

Retinoblastoma binding protein-1 (RBP1) is a member of the AT-rich interaction domain (ARID) superfamily, also known as ARID4A, that is involved in the repression of E2F-dependent transcription and cellular proliferation [5,6] through direct binding with the pocket domain of retinoblastoma (pRb) [7]. This repressive activity is largely due to a RBP1-mediated recruitment of mSin3A/histone deacetylase (HDAC) complexes to E2F-dependent promoters [8]. More recently, RBP1 deletion in mice was shown to result in a genomic imprinting defect at the Prader-Willi syndrome and Angelman syndrome imprinting center [9] and has been identified as a leukemia suppressor gene in mice [10]. Our laboratory has recently identified RBP1 as an estrogen-regulated gene in a U2OS osteosarcoma cell model expressing the estrogen receptors (ER) ERα and ERβ [11-13].

Although much is known regarding the function of RBP1 in pRb-mediated suppression of E2F activity, cellular proliferation, and in epigenetic modifications, nothing is known regarding the function of RBP1 in osteoblastic differentiation and function. Therefore, we investigated the effect of RBP1 suppression on osteoblastic differentiation in primary mouse calvarial osteoblasts. We found that suppression of RBP1 expression results in a delay in osteoblastic mineralization with a concomitant decrease in classic bone marker gene expression. Furthermore, our data demonstrates that RBP1 supports Runx2 expression through the use of the Runx2 P2 promoter and serves as a coactivator of Runx2 activity. This study identifies RBP1 as an important regulator of osteoblastic differentiation and provides a more comprehensive understanding of the osteogenic pathway in general.

## Methods

### Cell Culture and Reagents

Mouse calvarial osteoblast cultures were grown in Minimum Essential Media growth medium (αMEM; Invitrogen, Carlsbad, CA) supplemented with 1 × antibiotic/antimycotic (Invitrogen) and 10% (v/v) fetal bovine serum. The shRNA-stable mouse calvarial cell cultures, described in the text, were cultured in the same media supplemented with 5 mg/L blasticidin S (Boehringer Mannheim, Indianapolis, IN). For the osteoblast differentiation assays, αMEM growth medium supplemented with 50 mg/L ascorbic acid and 10 mM β-glycerophosphate, termed αMEM differentiation medium, was used. The human U2OS cell line was cultured in phenol red-free Dulbecco's Modified Eagle's Medium (DMEM)/F12 media containing 10% (v/v) fetal bovine serum (FBS) and 1 × antibiotic/antimycotic (Invitrogen). Ascorbic acid, β-glycerophosphate and Alizarin Red were purchased from Sigma-Aldrich (St. Louis, MO).

### Calvarial Osteoblast Preparation

One- to 3-day-old C57BL/6 mouse pups were sacrificed and the calvaria were minced and digested in Hank's balanced saline solution (Sigma-Aldrich) containing bovine serum albumin (4 mg/ml) and collagenase type 2 (4 mg/ml) for 10 min at 37°C. The first two digests were discarded since these mainly contain fibroblasts. The cells obtained from the third digest were resuspended in αMEM containing 10% (v/v) fetal bovine serum, expanded in culture dishes and subsequently used for the production of the CalOB-Control and CalOB-ΔRBP1 cell lines used in this study. The study was approved by the Mayo Clinic Institutional Animal Care and Use Committee (IACUC #A7909) and performed in accordance with internationally recognized ethical guidelines.

### shRNA Vector Construction and Stable-shRNA Cell Model Production

A lentiviral shRNA vector, termed pHREFBL/hU6 (+3276), containing a 21-mer siRNA sequence directed against +3299 to + 3320 relative to the transcriptional start site of mouse RBP1 (Accession # NM_001081195), GACGAGTCCCGAAGTATAAAG, was produced by America Pharma Source, LLC (Gaithersburg, MD). Expression of this construct is driven by a U6 promoter and contains a blasticidin S marker gene for positive selection of infected cells. A scrambled lentiviral control vector, containing the same nucleotide bases but in a random order, termed pHRBlasdU3-hU6, was also produced in the same manner. Both lentiviral vectors contain the loop sequence TTCAAGAGA. Primary mouse calvarial osteoblast cultures were plated in 24-well dishes at a cell density of 30-40% in MEM growth medium. The following day, 200 ul of either pHREFBL/hU6 (+3276) (RBP1 shRNA) or pHRBlasdU3-hU6 (Scrambled Control shRNA) lentiviral vector was added to each well and allowed to incubate at 37°C overnight. The viral stock media was then replaced with MEM growth medium for an additional 48 h. To select for stable lentiviral infection, 5 mg/L blasticidin S-containing growth medium was added to the cells for a 4 day selection period, changing the selection medium at 2 days. Each well was subcultured into T-75 culture flasks and the cells were maintained in αMEM supplemented with 5 mg/L blasticidin S. The resulting primary mouse calvarial shRNA cell cultures were termed CalOB-Control and CalOB-ΔRBP1. The experiments described in this manuscript were performed three times using independent calvarial cell preparations with similar results. Representative experiments are shown.

### Proliferation Assay

The CalOB-Control and CalOB-ΔRBP1 cell lines were seeded in growth medium into 96-well plates at a density of 6400 cells per well (2 × 10^4 ^cells/cm^2^) and allowed to proliferate (n = 6) for 72 hours. Twenty microliters of the Cell Titer 96 Aqueous One Solution Cell Proliferation Assay (Promega, Madison, WI) were added to each well and allowed to incubate at 37°C for 30 minutes. The plate was read at 490 nM on a SpectraMax 340 spectrophotometer (Molecular Devices Corp., Sunnyvale, CA) using the SoftMax Pro software (Molecular Devices Corp.).

### Differentiation Analyses and Alizarin Red Staining

The CalOB-Control and CalOB-ΔRBP1 cell lines were plated at approximately 50% confluence (1.3 × 10^4 ^cells/cm^2^) in 12-well dishes and allowed to reach confluence. At that time, αMEM differentiation media was added to the cells and changed every 3 days. Cells were harvested in triplicate for RNA at 0, 10, 17 and 31 days of culture using Trizol Reagent (Invitrogen). Parallel triplicate wells were stained for calcified bone nodules using Alizarin Red (Sigma-Aldrich). Briefly, cells were washed in 1 × PBS and fixed in 3.75% paraformaldehyde overnight at room temperature. Following two 1 × PBS washes, the cells were stained with 1.2% Alizarin Red (v/v) ph 4.2 for 20 min. The cells were extensively washed with 1 × PBS and subsequently scanned.

### Reverse Transcription PCR (RT-PCR) Analyses

Total RNA was harvested using TRIzol reagent following the manufacturers protocol (Invitrogen). One microgram (μg) of RNA was used in a reverse transcriptase (RT) reaction using the iScript cDNA Synthesis Kit (Biorad, Hercules, CA) according to manufacturer instructions. The RT reactions were diluted 1:5 and 2 μl used for real-time quantitative PCR (QPCR) using the Biorad iCycler (Biorad) and the iQ SYBR Green Supermix reaction kit (Biorad). Fold changes in gene expression were calculated as 2^(Housekeeping Ct - Gene Ct)^, where the housekeeping gene was TATA-binding protein (TBP). The primer sequences used in this study can be found in Table 1.

**Table 1:** Primer sequences for quantitative PCR (QPCR) used in this study

Gene/Symbol	Accession #	Orientation	Mouse-Specific Primer Sequences (5' to 3')
RBP1	NM_001081195	Forward	GCAAAAGACGAACCCCAAAGCGGAC
		Reverse	TCCTGCGTGAGACACTTCTCTGTC
Runx2	NM_009820	Forward	GCCGGGAATGATGAGAACTA
		Reverse	GGTGAAACTCTTGCCTCGTC
Osterix	NM_130458	Forward	GGAGGTTTCACTCCATTCCA
		Reverse	TAGAAGGAGCAAGGGGACAGA
BMP2	NM_007553	Forward	ACGTCCTCAGCGAATTTGAG
		Reverse	GCCTGCGGTACAGATCTAGC
Osteocalcin	NM_007541	Forward	GCCATCACCCTGTCTCCTAA
		Reverse	GCTGTGGAGAAGACACACGA
Alkaline Phos.	NM_007431	Forward	TGAGCGACACGGACAAGA
		Reverse	GGCCTGGTAGTTGTTGTGAG
BSP	NM_008318	Forward	TTCCCAGGTGTGTCATTGAAGA
		Reverse	GGTATGTTTGCGCAGTTAGCAA
TBP	NM_013684	Forward	GCACTTCGTGCAAGAAATGCTG
		Reverse	CATAGCTCTTGGCTCCTGTGC
Runx2: P1	N/A	Forward	CCGGCCACTTCGCTAACTT
		Reverse	TGGTGCTCGGATCTACAGGAA
Runx2: P2	N/A	Forward	TCTGGAAAAAAAAGGAGGGACTATG
		Reverse	GGTGCTCGGATCCCAAAAGAA

### Transient Transfection Analyses

U2OS or primary mouse calvarial osteoblast cells were plated at a density of 50% (2.6 × 10^4 ^and 1.3 × 10^4 ^cells/cm^2^, respectively) in 12-well plates the day before transfection. Two-hundred and fifty (250) ng of the p6OSE2-Luc reporter construct [14], or the mouse osteocalcin reporter construct [14], along with Runx2 [1] and RBP1 [15] expression constructs, were transiently transfected in triplicate using FuGENE 6 transfection reagent (Roche Diagnostics, Indianapolis, IN) for the U2OS cells or Lipofectamine LTX Reagent (Invitrogen) for the calvarial osteoblasts according to the manufacturer's instructions. Following incubation at 37°C for 48 hours, cells were harvested in 1 × Passive Lysis Buffer (Promega) and equal quantities of protein extracts were assayed using Luciferase Assay Reagent (Promega).

## Results

### Generation of a mouse calvarial osteoblast cell model with suppressed RBP1 expression

**Figure 1:**
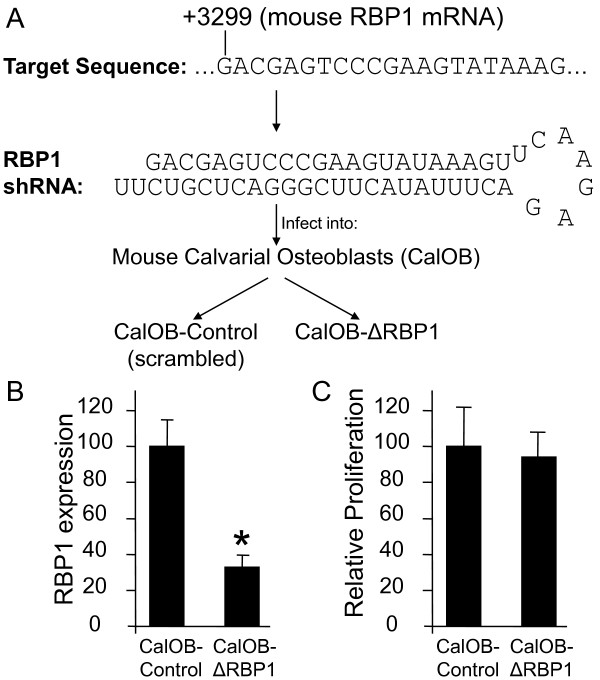
**shRNA design and confirmation of RBP1 knockdown in mouse calvarial osteoblasts**. **(A) **The sequence at +3299 to +3320 relative to the start of transcription of mouse RBP1 gene was selected and packaged into a shRNA lentiviral expression vector along with a scrambled shRNA control. Both lentiviral constructs were infected into wild-type mouse calvarial osteoblasts and stable cell lines were selected using blasticidin S for 2 weeks. The resulting cell lines are termed CalOB-Control and CalOB-ΔRBP1. **(B) **Total RNA from the CalOB-Control and CalOB-ΔRBP1 cell lines (n = 3) were isolated and processed for real-time QPCR analysis. The data are expressed as RBP1 expression relative to TBP controls and normalized to the CalOB-Control cell line. **(C) **The CalOB-Control and CalOB-ΔRBP1 cell lines were plated (n = 6), allowed to grow for 72 hours and a proliferation assay was performed. The data are expressed as relative cellular proliferation normalized to the CalOB-Control cell line. For all panels, the black bars represent the mean ± standard deviation. The asterisk represents statistical significance at the p < 0.01 level (ANOVA).

Numerous transcription factors are involved in the establishment and maintenance of osteoblastic differentiation such as Runx2, osterix and Dlx5 [16]. We have previously identified RBP1 as an estrogen regulated gene in U2OS osteosarcoma cells stably expressing ERα [11-13], however, no information exists on the function of RBP1 during the process of osteoblastic differentiation. As an initial step in understanding the role of RBP1 in this process, we generated a primary osteoblastic cell model in which RBP1 expression was reduced. A shRNA lentiviral expression vector was generated against the mouse RBP1 sequence and introduced into primary mouse calvarial osteoblasts (Figure 1A). A scrambled sequence control lentivirus was also generated and infected in an identical manner to serve as a control for non-specific effects of the viral infection. Following antibiotic selection, cell lines stably expressing the mouse RBP1 shRNA (CalOB-ΔRBP1) or the scrambled control shRNA (CalOB-Control) were established. Quantitative PCR (QPCR) analysis indicated a 65-70% reduction in RBP1 expression in CalOB-ΔRBP1 cells when compared to the CalOB-Control cell line (Figure 1B), demonstrating successful RBP1 suppression. A proliferation assay was performed on the CalOB-Control and CalOB-ΔRBP1 cell lines since RBP1 has been implicated in cell cycle regulation [5,6], however no difference in proliferative capacity of these cells was observed (Figure 1C).

### Effect of RBP1 suppression on osteoblastic differentiation and bone marker gene expression

**Figure 2:**
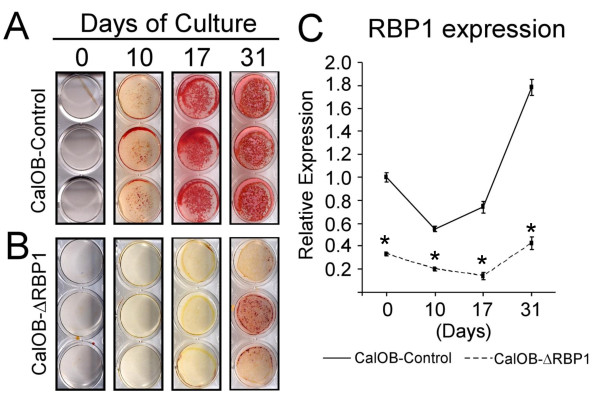
**Alizarin Red staining reveals a significant delay in osteoblastic mineralization of the CalOB-ΔRBP1 cell line**. The CalOB-Control **(A) **and CalOB-ΔRBP1 **(B) **cell lines were plated (n = 3) and allowed to differentiate in osteoblastic differentiation media for 0, 10, 17 and 31 days. The cells were subsequently stained using Alizarin Red. **(C) **Total RNA was extracted from identically treated CalOB-Control (solid line) and CalOB-ΔRBP1 (dotted line) cells and processed for real-time QPCR analysis (n = 3). The data are expressed as RBP1 expression relative to a TBP control and normalized to the day 0 CalOB-Control cell line. The data points represent the mean ± standard deviation. The asterisks represents statistical significance at the p < 0.01 level (ANOVA).

To determine the role of RBP1 in the establishment of the osteoblastic phenotype, the CalOB-ΔRBP1 and CalOB-Control cell lines were tested for the ability to form mineralized nodules when cultured in osteoblastic differentiation media for 10, 17 and 31 days of culture. As expected, mineralized bone nodules were observed following 10 days of culture in the CalOB-Control cell line with significant mineralization observed at 17 and 31 days of culture (Figure 2A). In contrast, only at 31 days of culture were mineralized nodules observed in the CalOB-ΔRBP1 cell line (Figure 2B), demonstrating a significant delay in the onset of osteoblastic differentiation following RBP1 suppression. Figure 2C shows that RBP1 expression remained low throughout the differentiation time-course in the CalOB-ΔRBP1 cell line. Interestingly, RBP1 expression increased approximately 1.8-fold during the course of differentiation in the control cell line suggesting that its expression is both regulated and important for this process.

**Figure 3:**
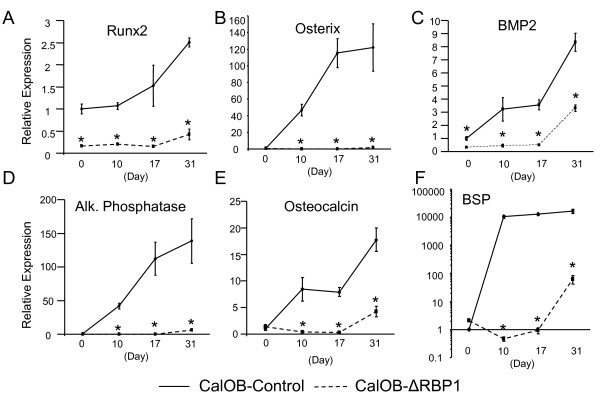
**Real-time QPCR analysis demonstrates suppressed bone marker gene expression in the CalOB-ΔRBP1 cell line**. Total RNA was extracted from CalOB-Control (solid line) and CalOB-ΔRBP1 (dotted line) cells treated as in Figure 2 and processed for real-time QPCR analysis (n = 3) for the indicated genes **(A-F)**. The data are expressed as target gene expression relative to TBP controls and normalized to the day 0 CalOB-Control cell line. The data points represent the mean ± standard deviation. The asterisks represents statistical significance at the p < 0.01 level (ANOVA).

Since RBP1 suppression resulted in a significant delay in mineralized nodule formation, we next determined whether bone marker gene expression followed a similar pattern of suppression. CalOB-ΔRBP1 and CalOB-Control cells were treated identically as in Figure 2 and analyzed for bone marker gene expression using QPCR. As shown in Figure 3A and 3B, the transcription factors Runx2 and osterix, both necessary for bone formation bone [1-4], are significantly suppressed in the CalOB-ΔRBP1 cell line and either do not increase, or only increase slightly during the differentiation process. Furthermore, as seen in Figure 3C-F, the classic bone marker genes bone morphogenetic protein-2 (BMP2), alkaline phosphatase, osteocalcin and bone sialoprotein (BSP), are also significantly suppressed in the CalOB-ΔRBP1 cell line. As with Runx2 and osterix, increased expression of these genes is only observed at the 31 day timepoint when the first signs of mineralization are observed in the CalOB-ΔRBP1 cell line (Figure 2B). This data unequivocally demonstrates that suppression of RBP1 expression in primary mouse calvarial osteoblasts results in a significant delay in osteoblastic mineralization with a concomitant suppression of bone marker gene expression. These data also reveal a novel and necessary role for RBP1 in the establishment of the osteoblastic phenotype.

### RBP1 regulation of Runx2 expression and transcriptional function

**Figure 4:**
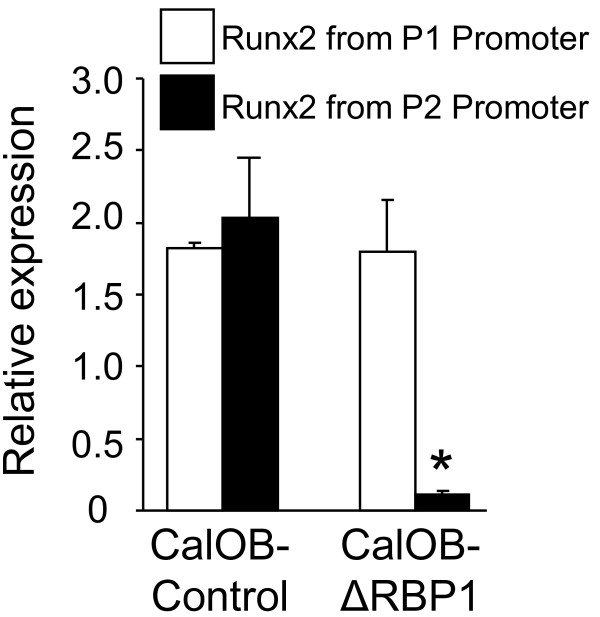
**RBP1 regulates Runx2 through the Runx2 P2 promoter**. Runx2-specific primer pairs designed to transcripts arising from either the Runx2 P1 promoter (open bars) or the Runx2 P2 promoter (closed bars) were used in a real-time QPCR analysis from RNA isolated from the CalOB-Control or CalOB-ΔRBP1 cell lines. The data are expressed as Runx2 expression relative to a TBP controls and normalized to the day 0 CalOB-Control cell line. The data represent the mean ± standard deviation. The asterisks represents statistical significance at the p < 0.01 level (ANOVA).

Runx2 is an essential mediator of the osteoblastic phenotype, as Runx2 deletion in mouse knockout models results in a complete elimination of mineralized bone [1-3]. Expression of Runx2 is controlled by two independent promoters, a distal P1 promoter and a proximal P2 promoter, which are largely regulated through distinct mechanisms [17]. Since suppression of RBP1 results in a significant decrease in Runx2 expression, we used primers specific for Runx2 transcripts arising from the P1 and P2 promoters in a QPCR analysis. As is shown in Figure 4, only transcripts arising from the P2 promoter, which are largely controlled through epigenetic mechanisms such as methylation [17], are suppressed following RBP1 depletion, demonstrating Runx2 promoter specificity in RBP1 function.

**Figure 5:**
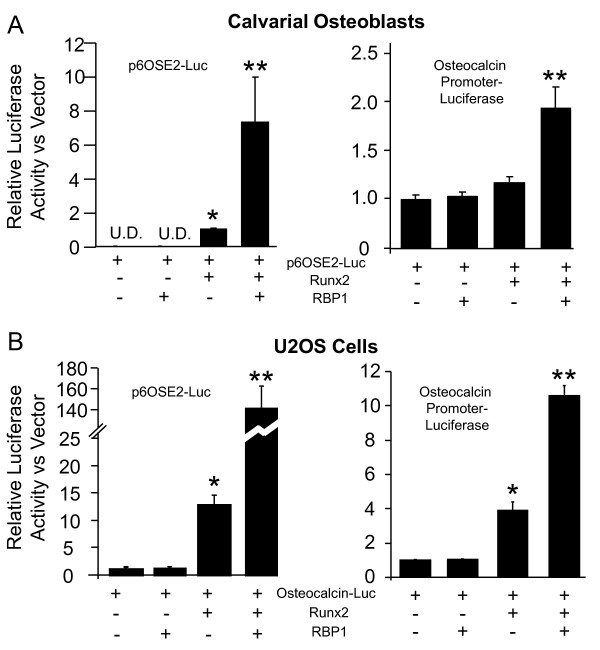
**RBP1 is a Runx2 coactivator in both U2OS osteosarcoma and mouse calvarial osteoblast cell models**. Mouse calvarial osteoblast **(A) **or human U2OS osteosarcoma **(B) **cells were transiently transfected (n = 3) with either the p6OSE2-Luc reporter construct or the mouse osteocalcin luciferase reporter construct as described under "Methods." The black bars represent the mean ± standard deviation. A single asterisk (*) represents significance of p < 0.01 (ANOVA) relative to the reporter construct alone and a double asterisk (**) represents significance of p < 0.01 (ANOVA) relative to the reporter and Runx2 expression construct alone. U.D. stands for undetectable.

Although little is known regarding the function of RBP1 in osteoblasts, its role as a retinoblastoma transcriptional coregulator is well known. Most of the members of the ARID transcription factor superfamily, including RBP1/ARID4A, weakly bind DNA without sequence specificity and primarily influence gene expression through protein-protein interactions [18]. Therefore, it is conceivable that RBP1 also acts as a coregulator of osteogenic transcription factors, such as Runx2. In order to assess the role of RBP1, if any, in coregulation of Runx2 activity, transient transfection analyses were conducted using two known Runx2-responsive luciferase reporter constructs, p6OSE2-Luc [14] and the mouse osteocalcin promoter [14]. As seen in Figure 5A, transient transfection of Runx2 alone into mouse calvarial osteoblasts resulted in a modest activation of the p6OSE2-Luc and the mouse osteocalcin reporter constructs, respectively. RBP1 alone did not alter the activity of either reporter construct; however coexpression of Runx2 and RBP1 resulted in a potent coactivation of both reporter constructs (8-fold and 2-fold). To verify the coactivation of Runx2 by RBP1 in an independent cell system, the same experiment was performed in the human U2OS osteosarcoma cell line (Figure 5B). As was observed in the mouse calvarial cells, expression of Runx2 alone resulted in a statistically significant 13-fold and 4-fold activation of the p6OSE2-Luc and the mouse osteocalcin reporter constructs. Coexpression of Runx2 and RBP1 resulted in a synergistic activation, 140-fold and 10.5-fold respectively, of the two reporter constructs. In combination, these data demonstrate that RBP1 not only regulates Runx2 expression, but also acts as a potent coactivator of Runx2 transcriptional function in two independent cell systems.

## Discussion

The objective of this study was to expand on our previous identification of RBP1 as a novel estrogen target gene in osteoblasts [11-13] by determining its role in the process of osteoblastic differentiation. We approached this interesting question through the production of a novel cell model where a lentiviral shRNA was stably introduced into primary mouse calvarial osteoblast cells. These cells exhibited a striking delay in the onset of mineralized nodule formation with a simultaneous suppression of bone marker genes such as Runx2, osterix, alkaline phosphatase, bone sialoprotein, osteocalcin and bone morphogenetic protein-2 (BMP2). This result is very similar to Runx2 -/- cells, which also exhibit deficiencies in the expression of these bone marker genes [2], suggesting that RBP1 may play a direct role in the normal expression and function of Runx2. This leads to the interesting notion that the osteogenic role of RBP1 is likely to be mediated, at least in part, by Runx2.

Runx2 is an important osteoblast lineage-determining transcription factor involved in directing precursor stem cells to the preosteoblast lineage and their concomitant differentiation [19]. Runx2 appears to be the master gene for osteoblast differentiation [14,20-22] as it induces the expression of osterix, a transcription factor which is required to finalize terminal osteoblast differentiation [4]. In addition, Runx2 plays a role in many other osteoblast functions such as activation of genes encoding osteocalcin, bone sialoprotein, osteopontin, and type 1 collagen and is also involved in autoregulation [2,3]. Mice lacking Runx2 and osterix have only a cartilaginous skeleton due to the lack of differentiated osteoblasts and die shortly after birth [1-4]. Furthermore, mutations in the Runx2 gene are found in patients with the human disorder Cleidocranial Dysplasia [23]. The data in this paper provide evidence that RBP1 plays an important role in regulating Runx2 expression and coactivation of Runx2-dependent bone marker genes, thus establishing RBP1 as an important mediator of Runx2 action in osteoblasts.

Recently a knock-out mouse model for RBP1 has been developed [9]. These mice display suppressed epigenetic modifications (e.g. chromatin/nucleosome methylation) at the PWS/AS genetic domain involved in Prader-Willi and Angelman Syndromes. In conjunction with this observation, we determined that RBP1 suppression decreases the amount of transcript arising from the Runx2 P2 promoter. Analysis of the Runx2 P2 promoter revealed a large CpG island extending from the P2 promoter to the 5' untranslated region of exon 1 of Runx2 [17], suggesting epigenetic modifications may be an important regulatory component of Runx2 P2-promoter-dependent activity. Another CpG island is located near the 3' end of the Runx2 gene [17], further suggesting that epigenetic modification may be an important regulatory facet of Runx2 regulation. This evidence demonstrating that RBP1 is involved in the modulation of epigenetic processes, along with our data demonstrating RBP1 regulation of the Runx2 P2 promoter, which is known to be regulated by epigenetic processes, is intriguing. Whether RBP1 directly regulates these processes at the Runx2 P2 promoter requires further experimentation.

## Conclusions

In its entirety, this paper presents evidence that the RBP1 transcription factor plays an important role in osteoblast biology since its suppression in primary mouse calvarial osteoblasts results in a striking delay in mineralization and suppression of well-known osteoblast marker genes. We further demonstrate that RBP1 regulates Runx2 expression through the proximal P2 promoter, a promoter known to be a target of epigenetic modifications, such as methylation. Interestingly, we also identified RBP1 as a potent transcriptional coactivator of Runx2 transcriptional activity. This underscores a dualism in the effects of RBP1 on Runx2, both through the maintenance of Runx2 expression as well as transcriptional coactivation. These important observations are the first to suggest a role for RBP1 in processes other than cell proliferation, and could open up novel approaches for the modulation of Runx2 activity in both *in vitro *and *in vivo *models of osteoblast function.

## Competing interests

The authors declare that they have no competing interests.

## Authors' contributions

DGM conceived of the study and was primarily responsible for its design, coordination, experimentation and drafting the preliminary manuscript. JRH, MS and TCS were responsible for the study design, direction, interpretation of the data and manuscript preparation. All authors have read and approved the final manuscript.

## Pre-publication history

The pre-publication history for this paper can be accessed here:

http://www.biomedcentral.com/1471-2474/11/104/prepub
